# Prevalence of Methicillin Resistant Staphylococcal Bioaerosols in and around Residential Houses in an Urban Area in Central India

**DOI:** 10.1155/2016/7163615

**Published:** 2016-01-26

**Authors:** P. Kumar, A. K. Goel

**Affiliations:** ^1^Biotechnology Division, Defence Research & Development Establishment, Gwalior 474002, India; ^2^Department of Microbiology, National Centre for Disease Control (NCDC), Delhi 110054, India

## Abstract

Methicillin resistant staphylococci (MRS) commonly found in clinical samples or associated environment pose a major health challenge globally. The carriage rate of MRS in human population is high, especially in India but research on airborne distribution of MRS is scanty. The present study aimed to evaluate the prevalence of MRS in indoor and outdoor environment of residential houses. Air samples were collected using impactor air sampler. The total counts of viable bacteria, staphylococci, and MRS along with the particles of various sizes were determined from indoor and outdoor environment of 14 residential houses. MRS bacteria were identified as methicillin resistant* S. aureus* (MRSA) or coagulase negative staphylococci (CNS) employing biochemical and PCR assays. The average concentration of MRS inside and outside of the houses was 5.9% and 4.6% of the total bacteria, respectively. The maximum correlation of total indoor and outdoor bacteria with particulate matter was 10 *μ*m (*r* = 0.74) and 5 *μ*m (*r* = 0.84), respectively. Statistically, significant positive correlation of staphylococci and MRS was found with particles of 10–25 *μ*m inside the houses. Molecular surveillance, antibiotic stewardship programme, and infection control policies can help to manage increasing MRS burden in developing countries.

## 1. Introduction

Bioaerosols and particulate matter of indoor and outdoor environment have a direct effect on the human health [1]. Bacteria have the diameter of about 2–8 *μ*m but these remain rarely free in the air. Generally, they tend to aggregate or attach to nonviable particles to form large clumps [2]. The total bacterial count is also significantly correlated with the number and size of particles [3, 4]. Staphylococci are quite hardy, nonspore forming, and relatively heat resistant commensal bacteria that can survive longer on dry and inanimate surface in every environment in which humans coexist. Their prevalence in the bioaerosols of residential environment is of great concern.

Methicillin resistant staphylococci (MRS) including* Staphylococcus aureus* (MRSA) and coagulase negative staphylococci (MR-CNS) can cause superficial to deep life-threatening diseases even in healthy or immune-compromised individuals [5]. Initially, MRSA infections were generally hospital-acquired (HA-MRSA) affecting the persons directly associated with healthcare facilities due to high risk or weak immune systems. Subsequently, MRSA infection spread to healthy people who have not been hospitalized and such strains were called community-associated MRSA (CA-MRSA). These are genetically distinguished from the HA-MRSA and may cause rapidly progressive and fatal disease like necrotizing pneumonia, severe sepsis, and necrotizing fasciitis [6]. MRSA is evolving continuously and now the distinction between CA-MRSA and traditional HA-MRSA is blurring [7].

In previous studies in India, carriage rate of MRS and involvement of MRSA in nosocomial infection was found higher than USA [8–10]. The studies revealed that more than 50% of people were nasal carriers for* S. aureus* alone and out of them 3.89% were positive for MRSA [11, 12]. Airborne MRSA may cause infection but their transmission frequency is lower than transmission via direct contact [13]. However, the airborne transmission has been implicated in a number of nosocomial outbreaks of MRSA [14, 15]. In a previous study, we have detected MRS in bioaerosols during a trade fair at Gwalior, India [16]. Thus, a method for direct isolation and identification of airborne MRS is desirable for molecular surveillance of MRS in countries having higher prevalence of these bacteria. The present study aimed to optimize a protocol for direct isolation of airborne MRS and to study their distribution in atmosphere of residential houses at Gwalior, Central India. Several studies have used the methods of impaction on agar media. However, in this study, we have used the antibiotics for the direct selection of methicillin resistant staphylococci (MRS) for the first time. Other researchers have first isolated the strains and then subjected to antibiotic susceptibility testing. This procedure requires one additional day to confirm whether it is methicillin resistant or sensitive.

## 2. Materials and Methods

### 2.1. Sample Collection

A total of 14 residential houses located in 7 different colonies (2 houses from each colony) were selected for indoor and outdoor bacterial aerosol sampling from Gwalior, Central India (longitude 78°13′E, latitude 26°13′N). Those houses were selected from the city that had no adverse health issues and the inhabitants were neither healthcare worker nor hospitalized within the past 1 year. Air samples for microbiological analysis were collected using Reuter Centrifugal Sampler (Biotest, Germany) at a height of 1.5 meters from the surface to simulate human breathing zone.

The head of air sampler was disinfected with alcohol swabs before each air sampling and the sampler was turned on for 2 min prior to sampling to allow the alcohol to evaporate. Sterile media strips containing microbial content test agar (MCA) supplemented with cycloheximide (100 *μ*g/mL), mannitol salt agar (MSA), and MSA containing 6 *μ*g/mL methicillin were loaded into the disinfected air sampler. Air samples were collected for 2 min in duplicate from inside and outside of each house at a flow rate of 280 L/min (the separation volume of the instrument was 40 L/min). MCA, MSA, and MSA containing methicillin were used for enumeration of total aerobic bacteria, staphylococci, and methicillin resistant staphylococci, respectively. The media strips were incubated at 35°C for 24 and 48 h to determine if the strips were overgrown. Air sampling results for cultivable bacteria were reported as colony forming units per cubic meter of air (CFU/m^3^) using the following formula:

(1) CFU/m3=Number  of  colonies  on  agar  strip×1000separation  volume×sampling  time  in  minutes.

Thus, a maximum of 1062 colonies were present in the strip. Each strip contains 34 compartments or wells, which means each well contains 31 colonies (1062/34 = 31).

### 2.2. Air Particulate Matter Measurement

Airborne particulate matter concentrations of six different sizes (aerodynamic diameter 0.3 *μ*m, 0.5 *μ*m, 1 *μ*m, 5 *μ*m, 10 *μ*m, and 25 *μ*m) were measured with commercial aerosol particle number counter (Lasair ll particle counter; Particle Measuring Systems, USA). Particles were counted in 4 sets (each set of 2 min) with 2 min delay after every set. Both particle counter and RCS sampler were operated simultaneously.

### 2.3. Biochemical Identification of MRS

Five representative colonies of mannitol fermenting and nonfermenting bacteria from MSA containing methicillin from each house (indoor and outdoor) were cultured on brain heart infusion (BHI) agar. Presumptive MRSA were further screened for coagulase and thermonuclease production. A part of colony was emulsified in normal saline then mixed with rabbit plasma; clumping indicated positive result [17]. Colonies grown on BHI agar were further incubated for 2 h at 60°C and overlaid with thermonuclease agar and further incubated at 37°C till the development of pink zone around the positive control [18].

### 2.4. PCR Identification of MRS

The bacterial colonies were grown in LB broth for 18 h. One mL of broth was centrifuged and the pellet was processed for DNA extraction using genomic DNA extraction kit as per the manufacturer instructions (MBI Fermentas, Vilnius, Lithuania). The amount and purity of the DNA were measured by spectrophotometer (NanoDrop-1000, Australia). A multiplex PCR was performed as described elsewhere, using the primers targeting a* Staphylococcus* specific region of the 16S rDNA,* S. aureus* specific* clf* gene encoding a surface-associated fibrinogen-binding protein, and* mecA* gene, a primary determinant of methicillin-resistance in both* S. aureus* (MRSA) and coagulase negative staphylococci (MR-CNS) species [19, 20].

### 2.5. Antimicrobial Susceptibility Testing

All the MRS isolates were subjected to antibiotic susceptibility testing by disc diffusion method according to Clinical Laboratory Standards Institute guidelines [21]. Fresh cultures from tryptic soy agar plate were picked up and suspended in PBS and the turbidity of the tube was adjusted as the 0.5 MacFarland standards. A 100 *μ*L of the suspension was swabbed evenly onto each Mueller-Hinton agar (MHA) plate (Difco laboratories, Sparks, MD). The agar surface was dried and the antibiotic discs were placed on the MHA surface using a sterile forceps. The disks were allowed to settle and the plates were incubated in inverted position at 37°C for 18–24 h. Macrolide, lincosamide, and streptogramin B (MLS) resistance phenotype were determined by placing erythromycin disc 20 mm away from clindamycin disc. The test was conducted in duplicate for each isolate and the organisms giving the same resistance profile in both the plates were included in the study.

### 2.6. Statistical Analysis

The statistical analysis was carried out using SigmaPlot 2000. Indoor and outdoor total bacterial count and staphylococcal and MRS count were compared using paired *t*-test. Relationship between bacterial counts and particle number was examined by Spearman correlation analysis.

## 3. Results and Discussion

### 3.1. Indoor and Outdoor Concentration of Bioaerosols (Bacteria, Staphylococci, and MRS)

The total bacterial concentrations inside the residential homes varied in the range of 5650 to 12425 CFU/m^3^ (8420.5 ± 2183.5). The outdoor total bacterial concentration ranged from 1775 to 13350 CFU/m^3^ (7405.4 ± 3142.4). The average indoor/outdoor (I/O) ratio was found to be 1.35 (Table 1). The difference between mean of indoor and that of outdoor total bacteria was nonsignificant (*P* > 0.05). Staphylococci concentration for indoor residential homes was found in the range of 1225 to 4125 CFU/m^3^ (2454.9 ± 880.96). Outdoor total bacterial concentration ranged from 700 to 2713 CFU/m^3^ (2074.3 ± 1037). The average I/O ratio was found to be 1.18. The difference between mean concentration of indoor and that of outdoor staphylococci was nonsignificant (*P* > 0.05). MRS concentration for indoor residential homes was found in the range of 162.5 CFU/m^3^ to 1175 CFU/m^3^ (495.7 ± 299.5). This was 5.9% of the total bacterial concentration. Outdoor total bacterial concentration ranged from 87.5 CFU/m^3^ to 806 CFU/m^3^ (338.03 ± 208.01) which was 4.6% of the total bacterial concentration. The average I/O ratio was found to be 1.7. The difference between mean indoor and outdoor concentration of MRS was statistically significant (*P* < 0.01, paired *t*-test) (Table 1).

The average concentration of airborne MRS was significantly higher inside the residential houses than outdoor environment (Table 1). The average concentration of staphylococci and total viable bacteria was also higher inside the residential homes but the differences between indoor and outdoor concentrations were statistically nonsignificant. The main contributor of indoor viable bacterial concentration is human activities including rafting, desquamated skin scales, and dry fabrics [4, 22]. Bed-making activities also liberate significantly higher concentration of MRSA in the air [23].* S. epidermidis* is a constituent of commensal microflora of the human skin and it is believed that pedestrians skin flora also contribute the elevated airborne staphylococcal concentration.

### 3.2. Correlation of Indoor and Outdoor Bioaerosols with Particulate Matter Size

Under indoor conditions, a significant positive correlation of total bacteria was found with particle size of 10 *μ*m (Spearman *r* = 0.74), followed by 5 *μ*m (*r* = 0.6) (Table 2). Significant correlation of staphylococci was found with particles of 10 *μ*m (*r* = 0.73), followed by 25 *μ*m (*r* = 0.59). However, MRS were correlated with particles of 25 *μ*m (*r* = 0.69), followed by 10 *μ*m (*r* = 0.54). Under outdoor conditions, the significant correlation of total bacteria was found with particles of size of 5 *μ*m (*r* = 0.84), followed by 10 *μ*m (*r* = 0.79), 1 *μ*m (*r* = 0.74), and 25 *μ*m (*r* = 0.64). Significant correlation of staphylococci was found with particles of 1 *μ*m (*r* = 0.72), followed by 5 *μ*m (*r* = 0.68), 0.5 *μ*m (*r* = 0.6), and 10 *μ*m (*r* = 0.6), whereas MRS were maximally correlated with particles of 10 *μ*m (*r* = 0.69), followed by 5 *μ*m (*r* = 0.78) and 25 *μ*m (*r* = 0.65).

The viable bacteria in air tend to aggregate and exist on large particles [2]. Staphylococci present in the indoor air are generally attached to desquamated skin scales and have the diameter range of 4–20 *μ*m. Skin fragments dispersed through the woven fabrics have a median size of 20 *μ*m [24]. In another study of bacteria recovered from skin fragments, 48% of bacteria were of 8.2 *μ*m or larger [25]. The significant positive correlations of indoor staphylococci including MRS with 10–25 *μ*m of particle in our results indicate the association of these bacteria with skin and nasal carriers (Table 2). The total indoor bacterial concentration was significantly correlated with particles 5–10 *μ*m in size.* S. aureus* on airborne particles have shown an average of four viable organisms per particle with a mean equivalent diameter of 14 *μ*m [24, 26]. Sneezing and coughing are also important sources of bioaerosols. In a study of relationship between viable bacteria and particle size, bacteria of >7.5 *μ*m correlated with exhaled carbon dioxide indicating nasal carriers [4]. Bacteria of size between 3 and 7.5 *μ*m (in tracheal and bronchial regions) remain in clumps whereas bacteria in the size range of 1–2 *μ*m (terminal bronchial region) remain free [4]. Our previous studies demonstrated a significant rise in airborne microbes including MRS during human gathering or anthropogenic activities in urban environment [16].

### 3.3. Multiplex PCR for Identification of MRS and Their Antibiotic Resistance Pattern

**Figure 1:**
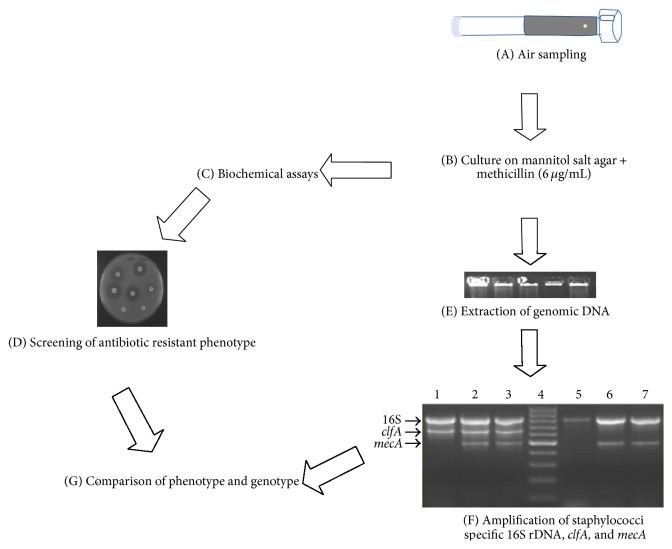
Flow diagram for isolation of MRS bioaerosols from residential houses. (F) Multiplex PCR for the detection of* mecA* gene in staphylococci. Lane 1:* S. aureus* ATCC 25923, lane 2: MRS isolate AMR 911, lane 3: MRS isolate AMR 927, lane 4: 100 bp ladder, lane 5:* S. epidermidis* ATCC 12228, lane 6: MRS isolate AMR 901, and lane 7: MRS isolate AMR 904.

The presumptive MRS strains selected from methicillin containing MSA strips were found to be PCR positive for staphylococci specific 16S rDNA gene. Out of them, 94% strains were found positive for* mecA* gene (Figure 1) and the remaining strains were observed to be PCR negative for* mecA* gene cassettes but were able to grow on methicillin concentration more than MIC. Among MRS, 9.5% of strains harbored* clf* gene specific for* S. aureus*.

A total of 87.4% of* mecA* positive (MRS) strains were found to be multidrug resistant (resistance for more than two different classes of antibiotics). MLS resistance phenotypes were found in 27.4% of strains including 16.7% of inducible expression (iMLS) and 10.7% of constitutive (cMLS) expression phenotype. The M resistance phenotype (resistance to erythromycin but not to lincosamides or streptogramins) was found only in 6 (7.1%) strains. Among the other antibiotics, 47.6% of strains were observed to be resistant to sulfamethoxazole-trimethoprim, 42.9% to quinolones, 8.3% to gentamicin, and 7.1% to mupirocin and 4.8% of strains were found to have intermediate resistance to tetracycline. All the MRS strains were found to be susceptible to linezolid and vancomycin.

MRS infections, including MRSA, occur most frequently among persons in hospitals and healthcare facilities who are at high risk or have weakened immune systems. Now CA-MRSA (USA300) has started replacing traditional MRSA (healthcare associated) in hospitals on a large scale and become dangerous epidemic strain worldwide [27]. The main objectives of this study were to optimize a methodology for direct isolation of airborne MRS and to determine their distribution pattern. Isolation of bioaerosols followed by their identification to genus (staphylococci) or species level and screening for the presence of antibiotic resistant determinant (*mecA* genotype) is a time consuming activity. In our previous report we did isolation of bacterial bioaerosols followed by their identification and characterization for* mecA* genotype which was labor intensive and required additional time [16]. Therefore, a direct method for isolation of airborne MRS is utmost important to complete a timely surveillance program.

MRS are well known as nasal colonizer in normal community and the colonization of virulent MRSA is significantly high [28, 29]. Airborne MRS may infect a person in two ways either by inhalation or by settling directly onto susceptible area, such as a wound [24]. MRS infections are important concern for health authorities and researchers across the world as it caused an approximate threefold increase in direct cost and prolonged hospital stay in comparison to infections due to methicillin sensitive staphylococci [30]. Infection of multidrug resistant MRS makes treatment very difficult because the drug of choice relies on newer generations of medicine which may be neither cost effective nor easily available to local market. Inappropriate and inadequate practices like misuse, abuse, and overprescription of antibiotics also result in the development of multidrug resistance [31]. Penicillin, cephalosporin, cotrimoxazole, and quinolones cover most of the antimicrobials prescribed in India [32]. Penicillin, cephalosporin, and quinolones are DNA damaging agents and can stimulate the drug resistance via SOS independent manner and through the induction of RecA-mediated repair, an error prone repair mechanism that induces mutations [33]. This repair accumulates various mutations which can lead to evolution in drug resistance.

Thus, the study confirmed that airborne MRS are commonly present in indoor and outdoor environment of the residential houses. Their concentration is significantly higher inside the houses as compared to the outdoor environment. Molecular surveillance, antibiotic stewardship programme, and infection control policies can help to manage increasing MRS burden in developing countries.

## Figures and Tables

**Table 1:** Bacterial concentrations from inside and outside of residential houses at Gwalior.

	Total bacteria			*S*. *aureus*			Methicillin resistant *S*. *aureus*		
	(CFU/m^3^)			(CFU/m^3^)			(CFU/m^3^)		
	Indoor	Outdoor	In/out	Indoor	Outdoor	In/out	Indoor	Outdoor	In/out
House 1	7088	5875	1.2	2050	1075	1.9	825	181.25	4.55
House 2	6275	5913	1.06	1575	1038	1.52	193.75	163.25	1.17
House 3	5650	4025	1.4	1475	1150	1.28	356.25	131.25	2.71
House 4	6925	1768	3.92	1225	700	1.75	275	87.5	3.14
House 5	7775	10175	0.76	1975	1713	1.15	662.5	400	1.66
House 6	7438	3950	1.88	1313	1100	1.19	275	212.5	1.29
House 7	12425	11013	1.13	3213	2350	1.37	881.25	618.75	1.42
House 8	11950	8900	1.34	2925	2313	1.26	1175	806	1.46
House 9	11275	13350	0.84	3050	3600	0.85	475	525	0.9
House 10	7375	8625	0.86	2568	3050	0.84	462.5	400	1.16
House 11	7413	6938	1.06	2825	2813	1	237.5	262.5	0.9
House 12	8725	6775	1.29	3475	1375	2.53	162.5	212.5	0.76
House 13	7175	6325	1.13	2575	3050	0.84	462.5	394	1.17
House 14	10413	10075	1.03	4125	3712.5	1.11	312.5	187.5	1.66

Average	8421.5 ± 2184.5	7407.64 ± 3142.4	1.35	2454.92 ± 880.96	2074.25 ± 1037	1.18	495.68 ± 299.47	338.04 ± 208.01	1.71^*∗∗*^

*P* value (95%)	*P* > 0.5			*P* > 0.5			*P* < 0.01		

^*∗∗*^Significant difference in indoor and outdoor MRS concentration (*P* < 0.01).

**Table 2:** Correlation between bacteria and particle size.

Correlation (Spearman *r*)		Particle size (number/m^3^)					
		0.3 *μ*m	0.5 *μ*m	1 *μ*m	5 *μ*m	10 *μ*m	25 *μ*m
Indoor	Total bacteria	−0.2967	0.09451	0.2352	0.6^*∗*^	0.736^*∗∗*^	0.4664
	Staphylococci	−0.2923	0.1516	0.244	0.4989	0.7319^*∗∗*^	0.5919^*∗*^
	MRS	−0.207	−0.2885	0.3216	0.4912	0.5352^*∗*^	0.6891^*∗∗*^

Outdoor	Total bacteria	−0.5297	0.2264	0.7363^*∗∗*^	0.8374^*∗∗∗*^	0.7934^*∗∗∗*^	0.644^*∗*^
	Staphylococci	−0.4224	0.6008^*∗*^	0.7217^*∗∗*^	0.677^*∗∗*^	0.5985^*∗*^	0.33
	MRS	−0.4361	0.1718	0.5176	0.7753^*∗*^	0.8261^*∗∗∗*^	0.6542^*∗*^

^*∗*^(*P* ≤ 0.05).

^*∗∗*^(*P* ≤ 0.01).

^*∗∗∗*^(*P* ≤ 0.001).
